# Murine Typhus with Renal Involvement in Canary Islands, Spain

**DOI:** 10.3201/eid1004.030532

**Published:** 2004-04

**Authors:** Michele Hernández-Cabrera, Alfonso Angel-Moreno, Evora Santana, Margarita Bolaños, Adela Francès, Antonio-Manuel Martín-Sánchez, Jose Luis Pérez-Arellano

**Affiliations:** * Hospital Universitario Insular de Gran Canaria, Canary Islands, Spain; † Universidad de Las Palmas de Gran Canaria, Canary Islands, Spain; 1 Drs. Hernández-Cabrera and Angel-Moreno contributed equally to the article.

**Keywords:** Rickettsia typhi, murine thypus, renal disease

## Abstract

Murine typhus and “murine-thypus-like” disease are reemerging infectious diseases. In Canary Islands (Spain), a rather distinct clinical pattern characterized by higher incidence of complications, especially renal damage (including acute failure and urinalysis abnormalities), is apparent and highly suggestive. It could be related to different strains of *Rickettsia typhi* or other cross-reactive species.

## The Study

Murine or endemic typhus is caused by *Rickettsia typhi*, formerly *R. mooseri* (*1*). Classic murine typhus is a zoonosis maintained in rats (*Rattus rattus* and *Rattus norvergicus*) and transmitted to humans through damaged skin by infected feces from the oriental rat flea (*Xenopsylla cheopis*) (*2*). New patterns of disease (“murine typhus-like” disease) have been described in recent years, and a new species of *Rickettsia* (*R*. *felis*) that causes a similar clinical picture has been identified (*1*–*3*). New modes of infection have been identified, including infection through inhalation of flea feces and transmission by different types of fleas (*Ctenophtephalis felis*) and from different reservoirs (e.g., dog, cat, and opossum).

Murine typhus occurs worldwide, particularly in warm and humid climates (*1*). In Spain, two seroepidemiologic surveys, in Salamanca and Madrid (Central/Western Spain), yielded seroprevalence rates of 12.8% and 68%, respectively, in the general population (*4*,*5*). However, no clinical cases have been reported. In Seville (Southwestern Spain), murine typhus is an important cause of fever of intermediate duration (*6*), and in Canary Islands, 10 autochthonous cases have been reported from Tenerife (*7*). For this reason, we include serologic testing for *R. typhi* in the evaluation of patients with fever of intermediate duration. We describe the clinical picture of murine typhus in the Canary Islands.

Adult (>14 years of age) in- and outpatients at the Hospital Universitario Insular of Las Palmas with a serologic diagnosis of murine typhus during December 1, 2000, through July 30, 2002, were included in our study. A case was defined by an immunoglobulin (Ig) M titer >1: 40, or a fourfold or higher increase in IgG titers against *R. typhi* by direct immunofluorescence test in 8 weeks (bioMeriéux, France), or both.

**Figure 1:**
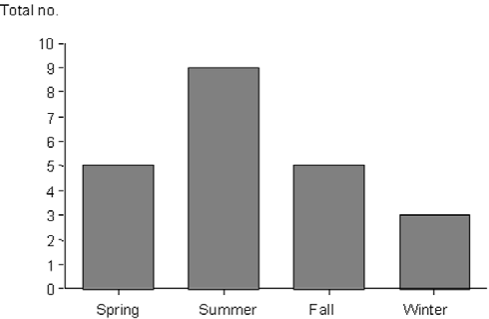
Distribution of cases of murine typhus by season.

**Figure 2:**
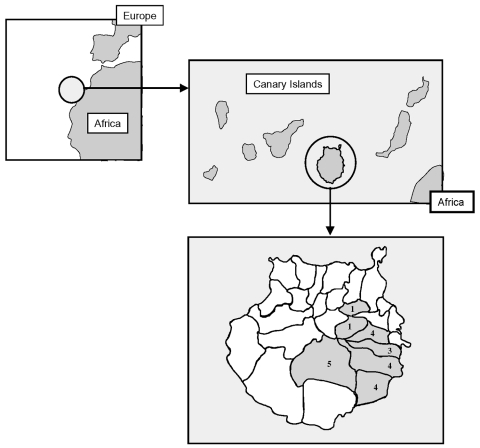
Distribution of cases of murine typhus by geographic area.

Epidemiologic, clinical, and laboratory data were collected. Antibodies against other agents that may cause a fever of intermediate duration (e.g., *Coxiella burnetii*, *R*. *conorii*, *Leptospira* sp., Epstein-Barr virus, and cytomegalovirus) were systematically tested. Twenty-two patients (21 men, 1 woman), with a mean age of 28 years (range 14 to 76 years), were included. Murine typhus was more frequent in summer (Figure 1). No case aggregation was observed. The geographic distribution is shown in Figure 2. All patients reported contact with animals (13 with dogs, 6 with horses, 5 with goats, 2 with cats, and 1 with camels).

The main symptoms and signs recorded are shown in Table 1. All patients had a high fever (mean 39.3°C) during a mean of 10 days (range 7 to 20 days). A light maculopapular, nonpurpuric rash, with rather centripetal distribution, was a frequent finding (68.2%). Up to one third of the patients had a dry cough. Hepatomegaly and, less frequently, splenomegaly were detected. Skin lesions suggestive of insect bites were found in 13.6% of the patients.

**Table 1:** Clinical findings^a^

Clinical findings	Location of study							
	Dumler, TX 1991	Silpapojakul, Thailand 1992	Bernabeu, Seville 1999	Fergie, TX 2000	Whiteford, TX 2001	Gikas, Crete 2002	Canary Islands 2003	
n	80	137	104	30	97	83	22	
Fever	98	100	100	100	100	100	100	
Headache	75	41	71	77	76	88	90	
Rash	54	20	62	80	63	80		
Arthromyalgia	46	44	77	57	40	45	45	
Hepatomegaly	-	24	29	-	-	22	38	
Cough	35	-	25	40	15	28	28	
Diarrhea	26	5	5	40	33	11	18	
Splenomegaly	-	5	24	-	-	23	14	
Bite	39	0	3	3,3	34	-	13	
Nausea/vomiting	48	3	23	43	45	18	13	
Abdominal pain	23	10	-	60	27	11	13	
Confusion	8	2	4	7	8	10	13	

^a^Data are expressed as a percentage; TX, Texas.

Four patients had a mild normocytic anemia. For most patients, leukocyte counts were normal, mild leukopenia was detected in two patients, and mild leukocytosis in four patients. Ten case-patients (45%) had thrombocytopenia. In most patients (89.5%), the erythrocyte sedimentation rate was high (11 mm to 83 mm), and the activated partial thromboplastin time (aPTT) was prolonged in six patients.

Aminotransferase elevation, usually four times above normal, was found frequently; two patients had normal values. Four patients had alanine aminotransferase values 10 times the normal value. Plasma bilirubin was normal for all patients.

In 36% of the patients, the plasma blood urea nitrogen was elevated; plasma creatinine was above normal in three cases (13%). In 19 cases (87%), alterations were found in the urinalysis. Fifteen patients had proteinuria and microhematuria with or without leukocyturia and granular casts, with a negative nitrite reaction. In two patients, isolated proteinuria occurred, and isolated microhematuria occurred in two other patients. All of these findings resolved quickly.

Eight patients fulfilled both diagnostic criteria (IgM >1:40 plus seroconversion), eight patients had initial IgM elevation, and six seroconverted without IgM increase. Cross-reactivity between *R*. *typhi* and other microorganisms was not observed. Fifty percent of the patients had serologic evidence of past infection with *C*. *burnetii* (12/22) or *R*. *conorii* (3/22) and, in one case, of co-infection with *C*. *burnetii*.

Eight cases were not treated because of spontaneous recovery. The remaining patients received doxycycline (100 mg twice a day). Fever disappeared from 1 to 6 days (median 2 days).

Three patients had severe signs and symptoms. Patient 6 was admitted with acute respiratory failure, lung infiltrates, and acute renal failure (plasma creatinine 2.8% mg), microhematuria, and leukocyturia. Intravenous fluids, doxycycline, ciprofloxacin, and methylprednisolone (1 g) were administered, and the patient rapidly improved. Autoantibodies and cryoglobulins were negative. Patient 16 had a dry cough and acute renal failure (plasma creatinine 2.7% mg) and later become disoriented. A cranial contrast computed tomography scan was normal, and cerebrospinal fluid (CSF) showed mononuclear pleocytosis (90 cells/μL), protein 70 mg/dL, and normal glucose. Doxicycline was administered with rapid neurologic improvement. Conjunctivitis and rash appeared but waned shortly after. Finally, patient 21 had a progressive meningeal syndrome, CSF showed mononuclear pleocytosis (19 cells/μL), increased protein (49 mg/dL), and normal glucose. The patient completely recovered in 48 hours under doxycycline.

Fever of intermediate duration has been defined by others in Spain as fever of 7 to 28 days without localizing signs (i.e., respiratory, digestive, urinary, or neurologic), plus the absence of diagnostic clues after a complete evaluation (*6*). A few diseases can account for most cases of this type of fever (mainly Q fever, brucellosis, boutonneuse fever, leptospirosis, mononucleosic syndromes, and murine typhus). In our area, autochthonous cases of boutonneuse fever or brucellosis have never been reported. Diagnosis is usually based on serology, which requires time for confirmation. Therefore, in the meantime, identifying clinical data for empirical treatment is important.

In our study, the number of cases per year is 12, higher than that in other areas of Spain (*6*), Israel (*8*), or the United States (*9*,*10*), with higher rate in summer. Most patients were male. All patients had direct contact with animals as reported by others (*9*,*10*); dogs were the most frequently cited animal (*9*,*10*).

The clinical features in our study are similar to those reported by others (*6*,*9*–*13*) with respect to those most frequent symptoms (fever, headache, and arthromyalgia) (Table 1). The incidence of rash is similar to that reported by Bernabeu (*6*) and Whiteford (*9*) and higher than that in other series. Reports of insect bites were more frequent in our study than studies from other areas (Bernabeu [*6*] and Silpapojakul [*11*]), but more insect bites were reported from a Texas study (*9*).

The laboratory findings in our study are similar to findings in other studies, although its relative frequency is variable (Table 2). The frequency of anemia varies from 1% to 69%, leukopenia from 4% to 40%, and thrombocytopenia from 3% to 60% (*6*,*9*–*11*). While 80% of the patients with Q fever in our area have a prolonged aPTT, 27% of the patients with murine typhus displayed this abnormal coagulation test. An elevation of aminotransferases in the range of viral hepatitis was common, but hyperbilirubinemia is exceptional and usually associated with alcoholism, co-infection, or glucose-6-phosphate dehydrogenase deficiency.

**Table 2:** Comparative laboratory findings in patients with murine typhus^a^

Laboratory test	Location of study							
	Dumler, TX	Silpapojakul, Thailand	Bernabeu, Sevilla	Fergie, TX	Whiteford, TX	Gikas, Crete	Canary Islands	
Anemia	75	-	1	57	69	25	18	
Leukopenia	28	4	18	40	37	7	9	
Leukocytosis	29	-	20	3	1	0	18	
Neutrophilia	-	-	-	63	77	-	36	
Thrombocytopenia	48	3	19	60	43	51	45	
ESR elevated	-	-	59	75	81	-	89	
Increased ratio prothrombin time	30	-	-	-	-	-	23	
Increased ratio aPTT	-	-	-	-	-	-	27	
Plasma BUN increased	27	-	-	0	3	-	36	
Plasma creatinine increased	21	-	-	0	0	-	14	
Hyponatremia	60	-	-	66	58	37	18	
Plasma creatine kinase increased	21	-	-	-	-	42	10	
Plasma LDH increased	87	-	-	-	100	82	81	
Plasma AST increased	90	-	67	67	82	86	77	
Plasma ALT increased	73	-	67	67	38	64	99	
Plasma alkaline phosphatase increased	60	-	25	-	-	15	30	
Plasma GGT increased	-	-	-	-	-	-	57.2	
Hypoalbuminemia	89		-	46	87	82	54.5	
Hypergammaglobulinemia	-		-	-	-	-	75.0	

^a^Data are expressed as a percentage. TX, Texas; ESR, erythrocyte sedimentation rate; aPTT, activated partial thromboplastin time; BUN, blood urea nitrogen; LDH, lactate dehydrogenase; AST, aspartate aminotransferase; ALT, alanine aminotransferase; GGT, gamma glutamic transferase.

Nephrologic alterations had a high frequency in our study. Three patients had acute renal failure, and 87% had some abnormality in the urinalysis, mainly microhematuria. These data are in sharp contrast with the low incidence of urinary alterations found in other studies. Some broad studies (*6*,*9*,*10*) do not report urinary abnormalities in murine typhus, though Dumler et al. (*13*) reported microhematuria or proteinuria in 28% of their patients. In a study specifically focused on renal involvement in murine typhus, Shaked et al. observed urinary abnormalities in 5 of 27 patients studied (*8*). To the best of our knowledge, 11 cases of acute renal failure have been related to *R*. *typhi* (*9*,*11*,*14*,*15*).

In general, murine typhus is a mild disease. However, a number of miscellaneous complications have been described. Our severe cases accounted for 13% (one renopulmonary syndrome, one encephalitis, and one meningitis with renal failure).

## Conclusions

In summary, in Canary Islands, incidence of murine typhus seem to be higher, patients more frequently report contact with dogs, the frequency of complicated cases is higher, and the incidence of renal involvement is higher. These data define a clinical picture of murine typhus that is somewhat different for the Canary Islands. These differences could be attributed to age (infantile versus adult series), mode of transmission or infection, or different strains of *R. typhi*. The diagnostic methodology was indirect, so cross-reaction with other rickettsiae cannot be excluded (*11*). Moreover, in our area, dogs are frequently parasitized by the flea of cats, a well-known vector for *R. felis* (*3*). More studies with direct diagnostic methods are needed to better define these differences. Finally, detecting urinary abnormalities in the setting of fever of intermediate duration, especially if associated with skin rash, thrombocytopenia, or hypertransaminasemia, in our geographic area is strongly suggestive of murine typhus.
