# Correction Vol. 10, No. 3

**DOI:** 10.3201/eid1007.C211007

**Published:** 2004-07

**Keywords:** errata, erratum, correction

In the article “Murine Typhus with Renal Involvement in Canary Islands, Spain” by Michele Hernández-Cabrera et al., errors occurred in the 2nd paragraph under The Study on page 740: 68% should be 6.8%. The corrected sentence appears below:

In Spain, two seroepidemiologic surveys, in Salamanca and Madrid (Central/ Western Spain), yielded seroprevalence rates of 12.8% and 6.8%, respectively, in the general population (4,5).

The corrected article appears online at http://wwwnc.cdc.gov/eid/article/10/4/03-0532_article.htm.

We regret any confusion this error may have caused.

