# Correction, Vol. 10, No. 4

**DOI:** 10.3201/eid1009.C11009

**Published:** 2004-09

**Keywords:** errata, erratum, correction

Correction heading for “Murine Typhus with Renal Involvement in Canary Islands, Spain” by Michele Hernandez-Cabrera et al. was inaccurate. Article appeared in Vol. 10, No. 4 online at http://wwwnc.cdc.gov/eid/article/10/4/03-0532_article.htm.

We regret any confusion these errors may have caused.

